# Supplementary material for: Inhibition of GCN2 Reveals Synergy with Cell-Cycle Regulation and Proteostasis
Source: Metabolites. 2023 Oct 9;13(10):1064. doi: 10.3390/metabo13101064 (PMC10609202; doi:10.3390/metabo13101064)
Supplement: Supplementary file 1 [file metabolites-13-01064-s001.zip › Figure S1.pdf]

Figure S1: Dose-response curves for experimental anticancer drugs.

Cell growth was monitored using the IncuCyte system (Essen Biosciences). An end-point approach was adopted whereby cell confluence was measured at the end of the assay. Cells were seeded in 96-well flat-bottom plates at 3000 cells/well in growth medium and allowed to attach overnight. The next day, the growth medium from each well was replaced with 300 µL of growth medium with or without treatment. Cells were stored in the cell incubator until untreated control cells reached >90% confluence, upon which confluence was recorded in each well. IC_{50} curves were plotted from data obtained in this assay using the logistic growth equation.

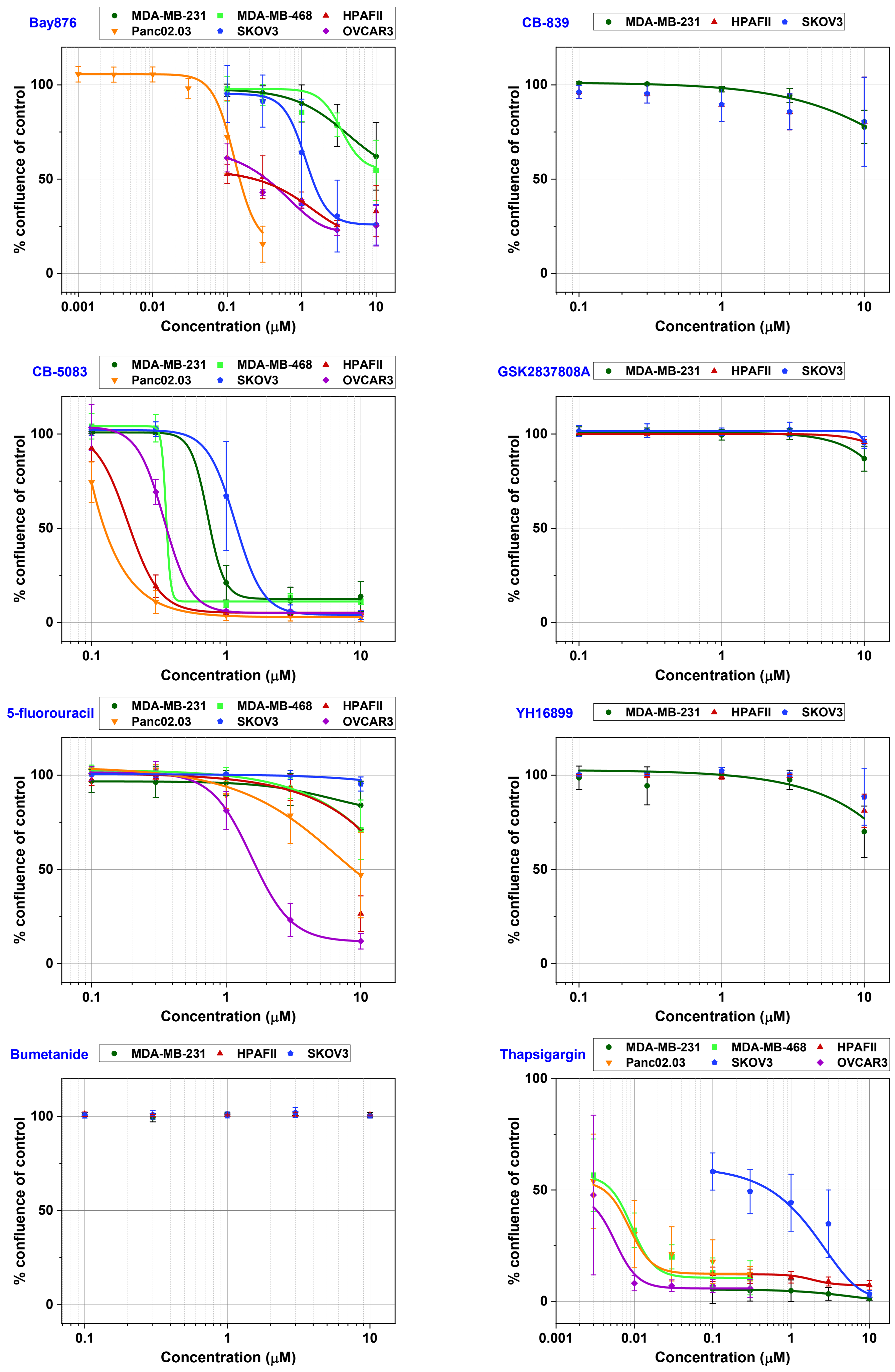
Bay876
MDA-MB-231
MDA-MB-468
HPAFII
Panc02.03
SKOV3
OVCAR3
% confluence of control
Concentration (μM)
CB-839
MDA-MB-231
HPAFII
SKOV3
% confluence of control
Concentration (μM)
CB-5083
MDA-MB-231
MDA-MB-468
HPAFII
Panc02.03
SKOV3
OVCAR3
% confluence of control
Concentration (μM)
GSK2837808A
MDA-MB-231
HPAFII
SKOV3
% confluence of control
Concentration (μM)
5-fluorouracil
MDA-MB-231
MDA-MB-468
HPAFII
Panc02.03
SKOV3
OVCAR3
% confluence of control
Concentration (μM)
YH16899
MDA-MB-231
HPAFII
SKOV3
% confluence of control
Concentration (μM)
Bumetanide
MDA-MB-231
HPAFII
SKOV3
% confluence of control
Concentration (μM)
Thapsigargin
MDA-MB-231
MDA-MB-468
HPAFII
Panc02.03
SKOV3
OVCAR3
% confluence of control
Concentration (μM)

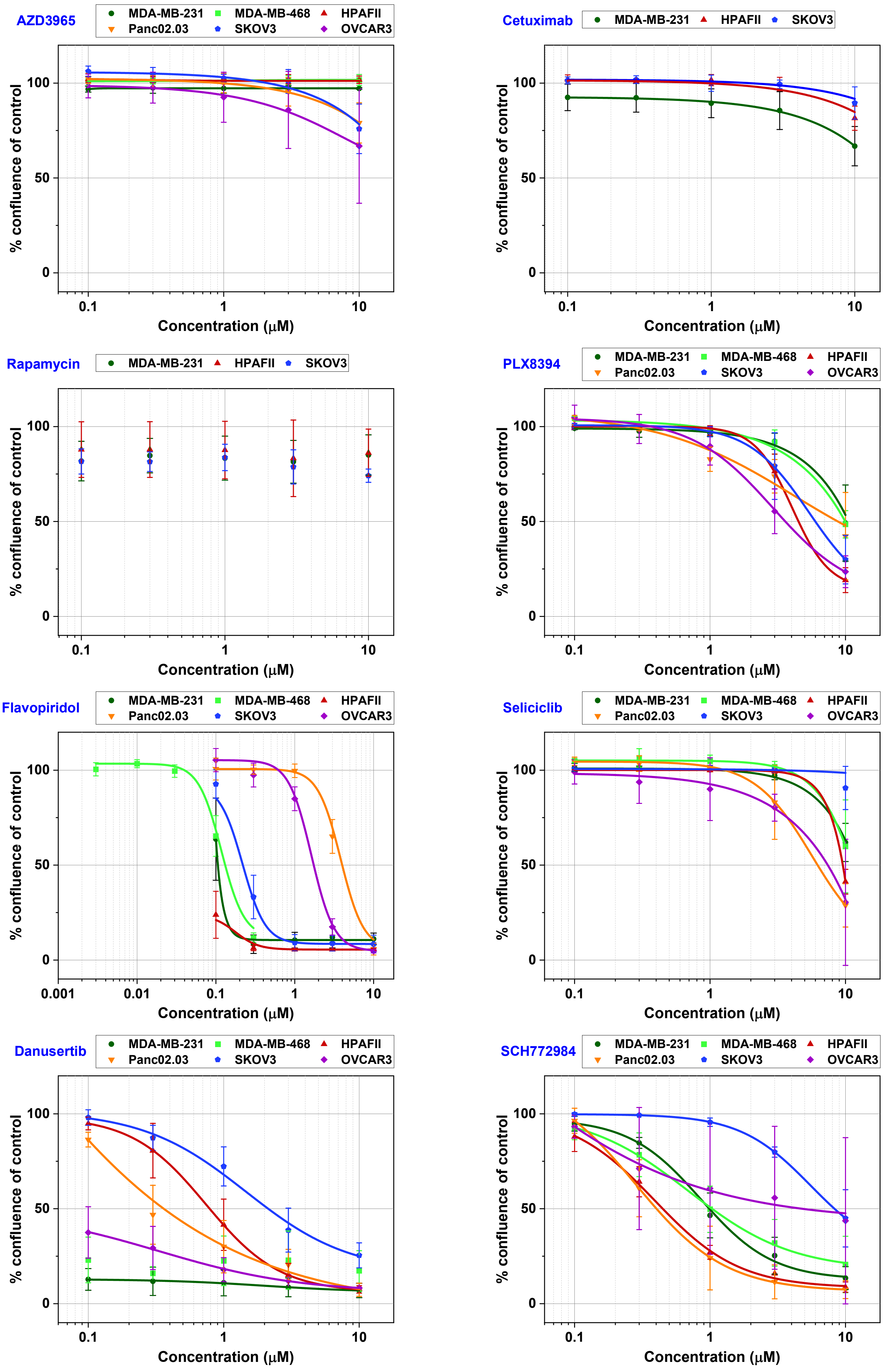
AZD3965
MDA-MB-231
MDA-MB-468
HPAFII
Panc02.03
SKOV3
OVCAR3
% confluence of control
Concentration (µM)
Cetuximab
MDA-MB-231
HPAFII
SKOV3
% confluence of control
Concentration (µM)
Rapamycin
MDA-MB-231
HPAFII
SKOV3
% confluence of control
Concentration (µM)
PLX8394
MDA-MB-231
MDA-MB-468
HPAFII
Panc02.03
SKOV3
OVCAR3
% confluence of control
Concentration (µM)
Flavopiridol
MDA-MB-231
MDA-MB-468
HPAFII
Panc02.03
SKOV3
OVCAR3
% confluence of control
Concentration (µM)
Seliciclib
MDA-MB-231
MDA-MB-468
HPAFII
Panc02.03
SKOV3
OVCAR3
% confluence of control
Concentration (µM)
Danusertib
MDA-MB-231
MDA-MB-468
HPAFII
Panc02.03
SKOV3
OVCAR3
% confluence of control
Concentration (µM)
SCH772984
MDA-MB-231
MDA-MB-468
HPAFII
Panc02.03
SKOV3
OVCAR3
% confluence of control
Concentration (µM)

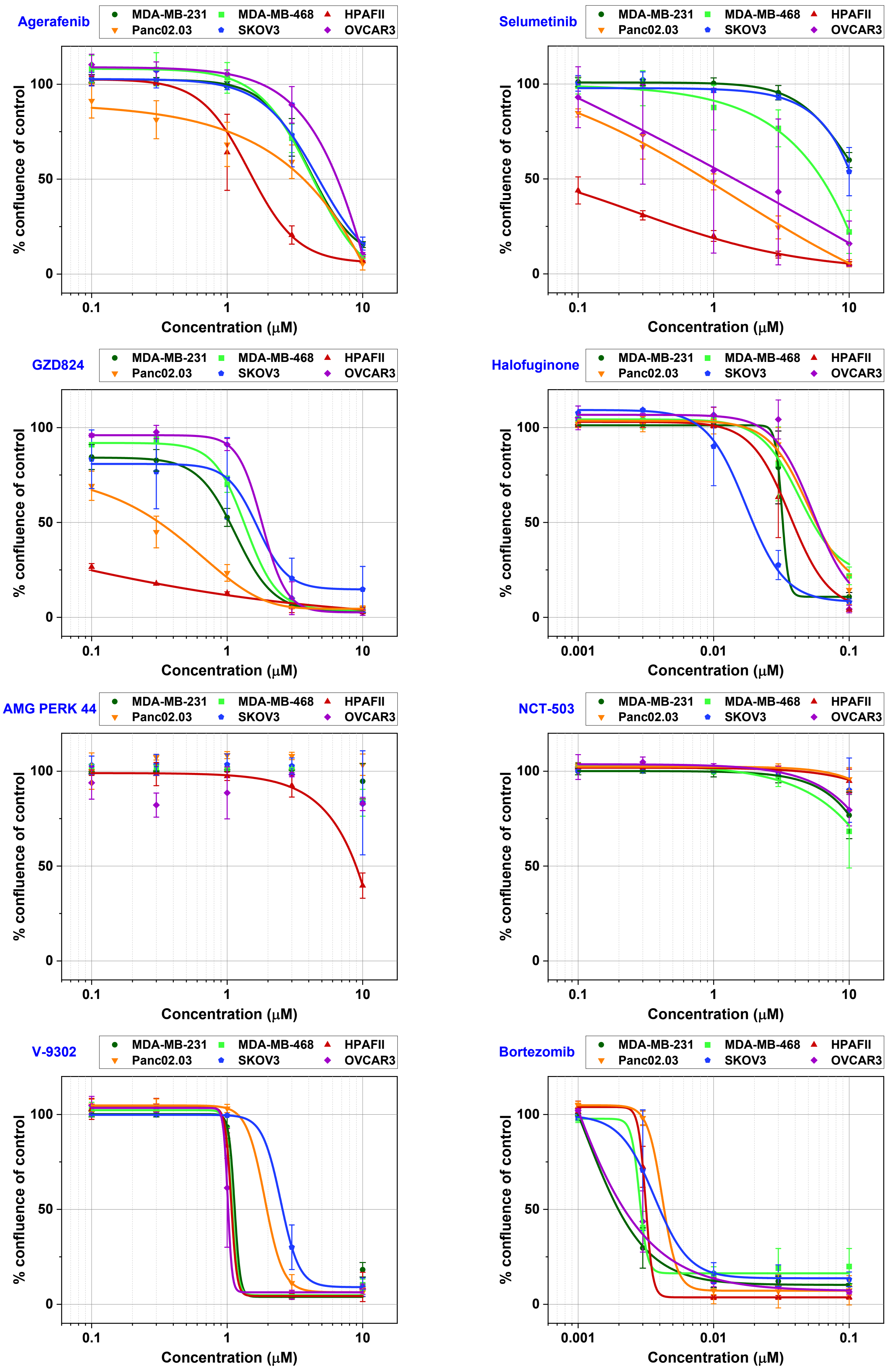

Agerafenib
Selumetinib
GZD824
Halofuginone
AMG PERK 44
NCT-503
V-9302
Bortezomib
MDA-MB-231
MDA-MB-468
HPAFII
Panc02.03
SKOV3
OVCAR3
% confluence of control
Concentration (μM)
0
50
100
0.1
1
10
0.001
0.01
