# Supplementary material for: Inhibition of GCN2 Reveals Synergy with Cell-Cycle Regulation and Proteostasis
Source: Metabolites. 2023 Oct 9;13(10):1064. doi: 10.3390/metabo13101064 (PMC10609202; doi:10.3390/metabo13101064)
Supplement: Supplementary file 1 [file metabolites-13-01064-s001.zip › Figure S2.pdf]

Figure S2 Evaluation of synergism

- Experimental and approved drugs (EAD) at the concentration listed in Table 2 were incubated with cells alone or in combination with 3 µM TAP20. The effect of TAP20 alone is indicated by the red dashed line. Confluence was measured in all wells when controls reached >90% confluence. Significant differences of growth are indicated by * ($p < 0.05$), ** ($p < 0.01$) and *** ($p < 0.001$).

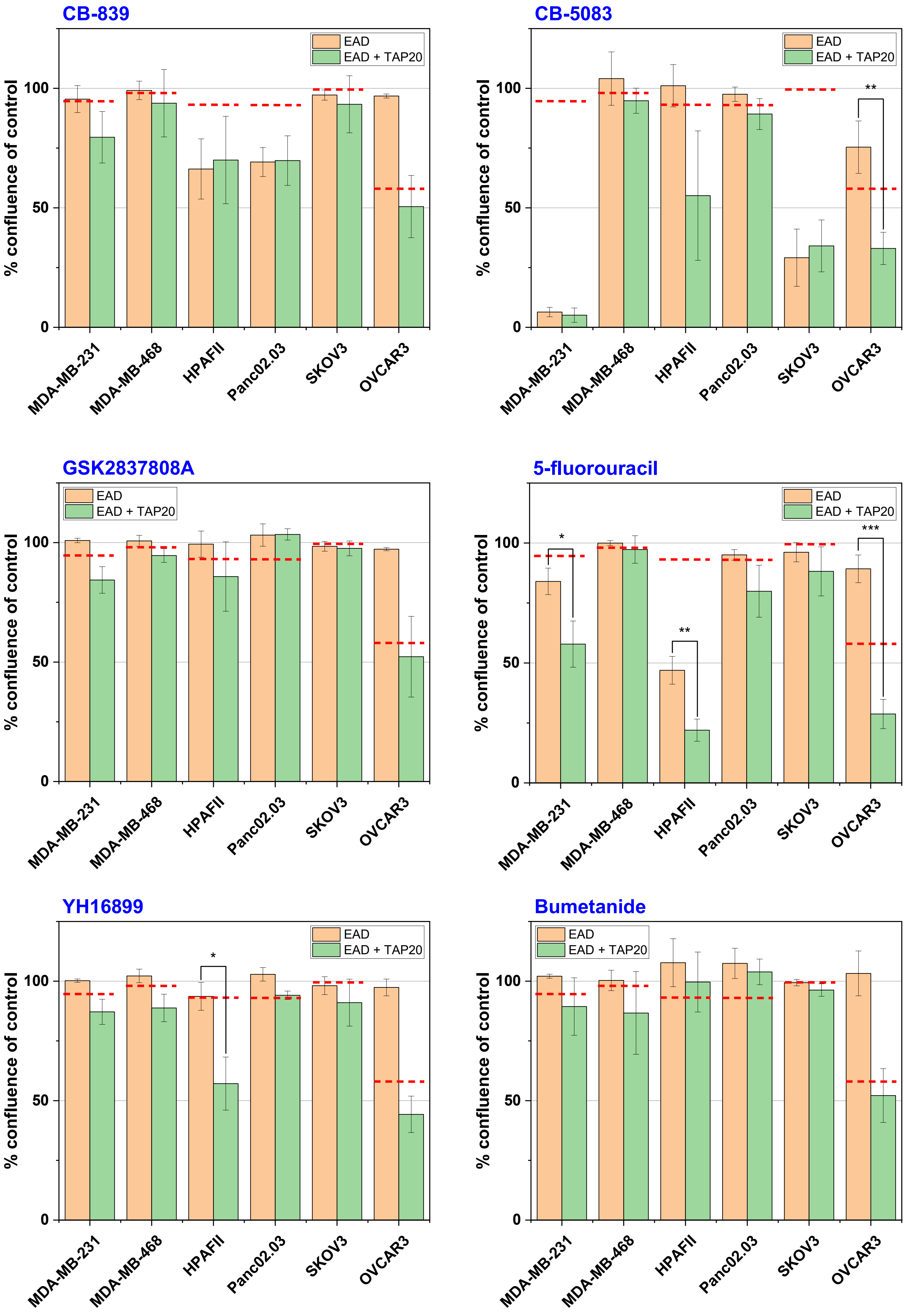

CB-839
CB-5083
GSK2837808A
5-fluorouracil
YH16899
Bumetanide
EAD
EAD + TAP20
% confluence of control
0
50
100
MDA-MB-231
MDA-MB-468
HPAFII
Panc02.03
SKOV3
OVCAR3
*
**
***

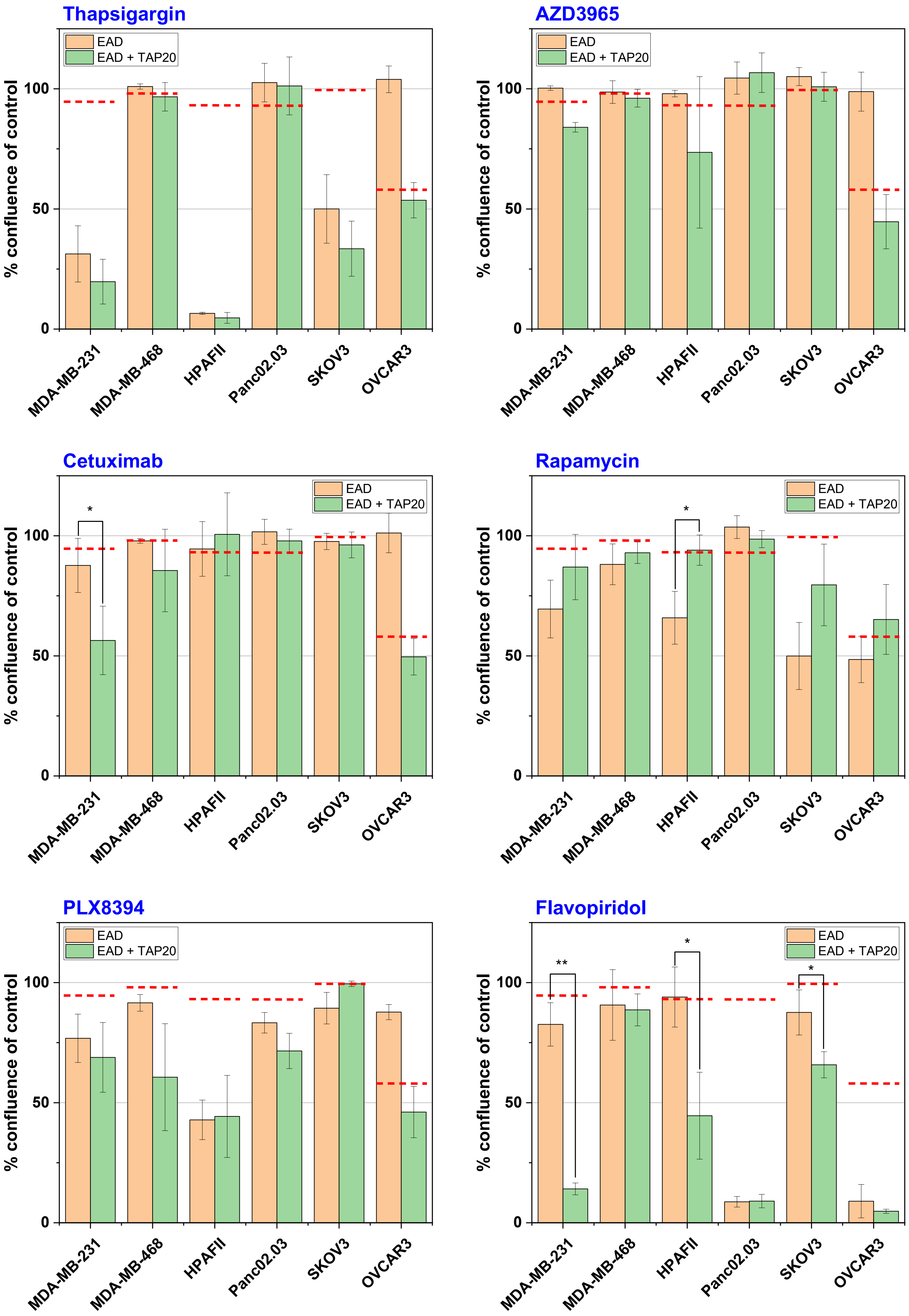
Thapsigargin
AZD3965
Cetuximab
Rapamycin
PLX8394
Flavopiridol
EAD
EAD + TAP20
% confluence of control
0
50
100
MDA-MB-231
MDA-MB-468
HPAFII
Panc02.03
SKOV3
OVCAR3
*
**

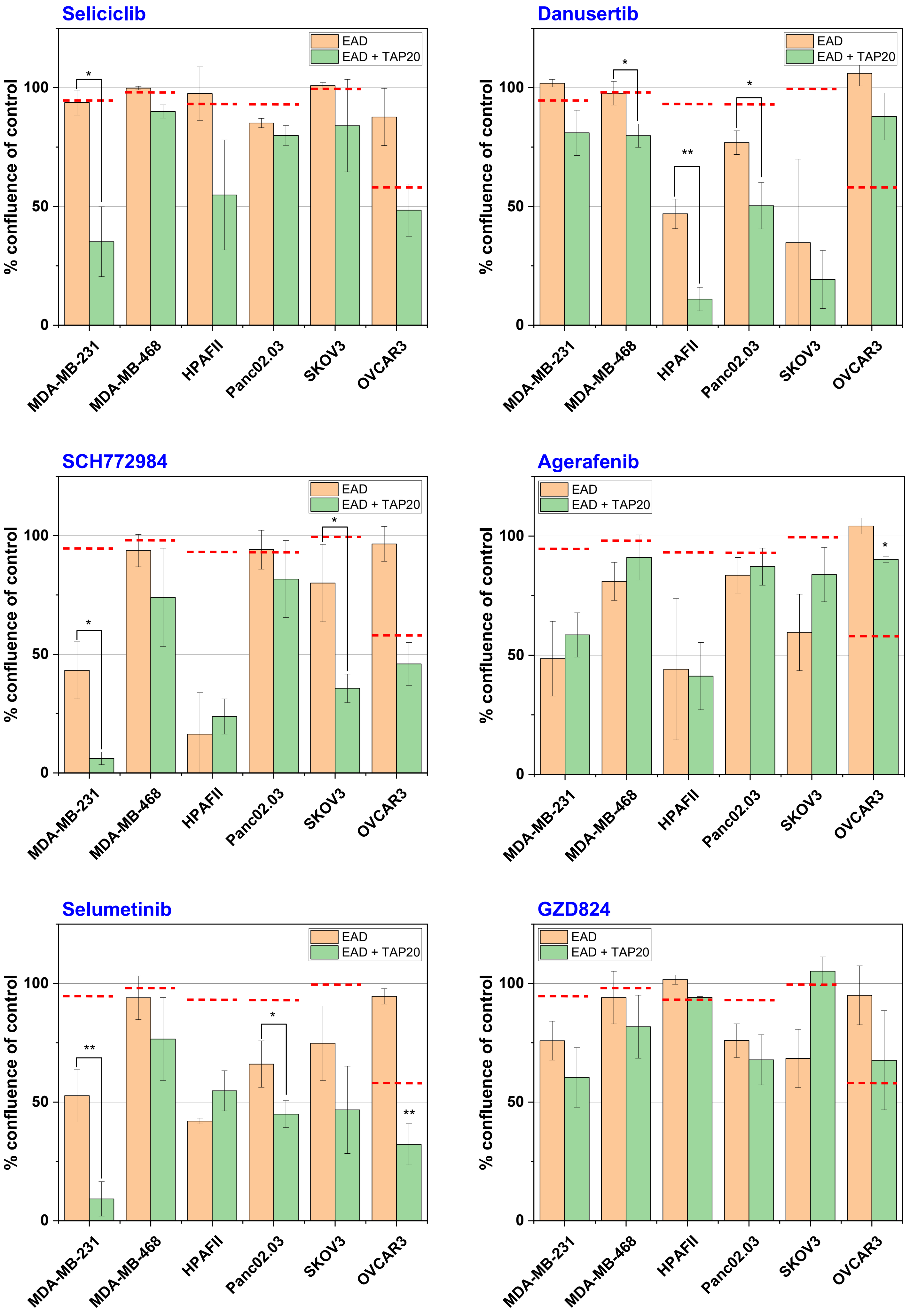
Seliciclib
Danusertib
SCH772984
Agerafenib
Selumetinib
GZD824
EAD
EAD + TAP20
% confluence of control
0
50
100
*
**
MDA-MB-231
MDA-MB-468
HPAFII
Panc02.03
SKOV3
OVCAR3

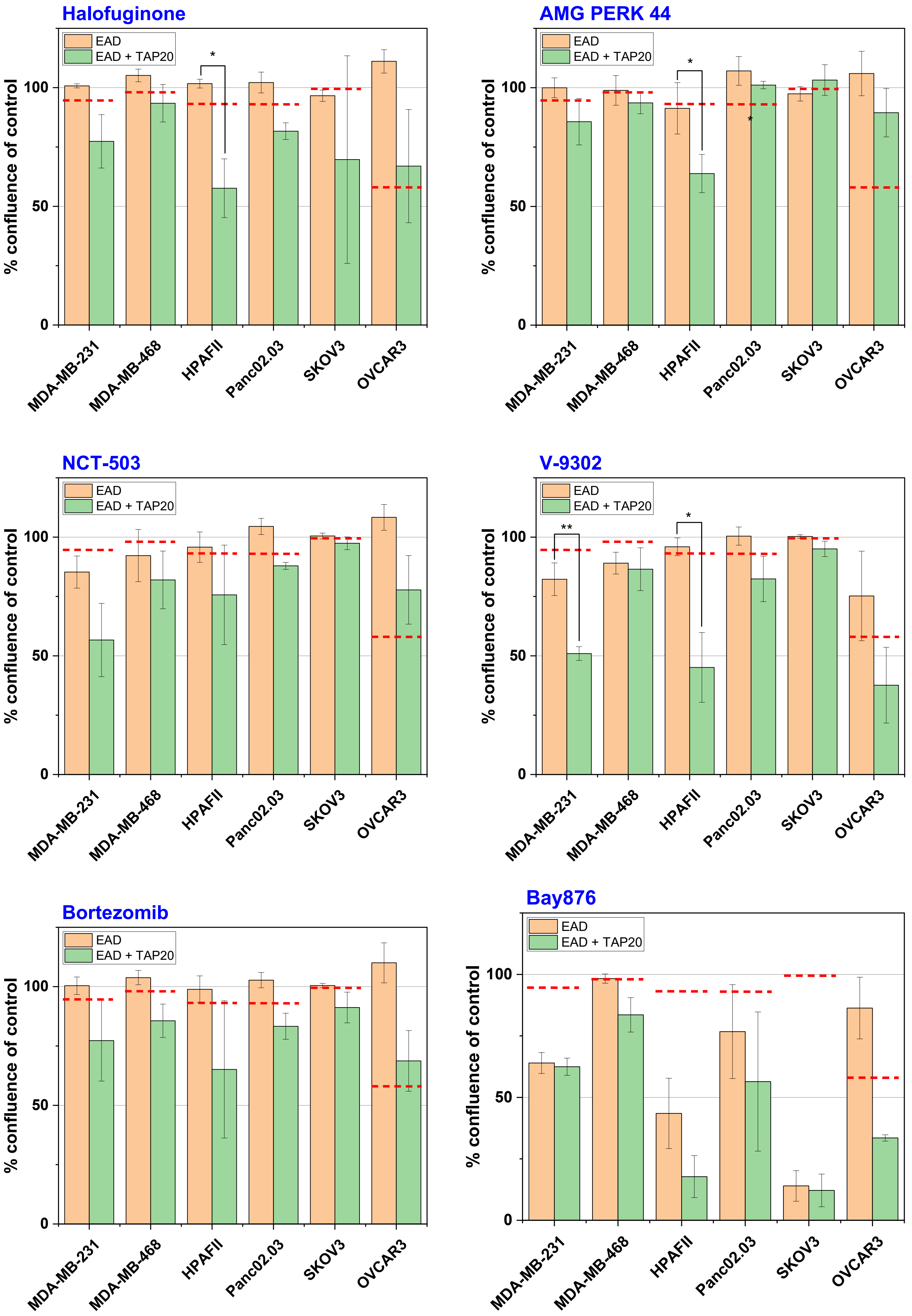

Halofuginone
AMG PERK 44
NCT-503
V-9302
Bortezomib
Bay876
EAD
EAD + TAP20
% confluence of control
0
50
100
*
**
MDA-MB-231
MDA-MB-468
HPAFII
Panc02.03
SKOV3
OVCAR3
